# The Resistance Abilities of Some *Bacillus* Species to Gastrointestinal Tract Conditions: Whole Genome Sequencing of the Novel Candidate Probiotic Strains *Bacillus clausii* BA8 and *Bacillus subtilis* BA11

**DOI:** 10.1002/fsn3.70018

**Published:** 2025-02-05

**Authors:** Nursel Söylemez‐Milli, Pelin Ertürkmen, Duygu Alp Baltakesmez

**Affiliations:** ^1^ Scientific Industrial and Technological Application and Research Center Bolu Abant Izzet Baysal University Bolu Turkey; ^2^ Department of Food Processing, Vocational School of Burdur Food, Agriculture and Livestock BurdurMehmet Akif Ersoy University Burdur Turkey; ^3^ Department of Gastronomy and Culinary Arts, School of Tourism and Hospitality Management Ardahan University Ardahan Turkey

**Keywords:** *Bacillus* spp., GI tract, MALDI‐TOF MS, probiotic, whole genome analysis

## Abstract

This study aims to investigate the resistance of potential probiotic *Bacillus* species to various conditions in the gastrointestinal (GI) tract and their safety characteristics. MALDI‐TOF MS identified all tested strains with a good safety score of ≥ 2.0; the strains demonstrated the capacity to pass through the Gl tract, exhibiting a reduction of > 6 log/CFU live cells. Furthermore, they exhibited varying survival rates in an acidic environment (pH 2.0–3.0) and the presence of Ox‐Bile (1% w/v) (*p* < 0.05). Following exposure to pH 3.0 and Ox‐Bile, the survival rate of *Bacillus* spp. ranged between 85.94% and 91.24% and 87.30% and 91.54%, respectively. The results of the in vitro experiments showed that the six *Bacillus* strains had comparable characteristics (e.g., tolerance to GI track enzyme, auto‐aggregation ability) to the reference probiotic strain *Lactiplantibacillus plantarum* LA15. The auto‐aggregation results of the *B. clausii* BA8 strain, which has demonstrated resistance to GI tract conditions, were also noteworthy. This strain showed 72.32% after 2 h and 74.55% at the end of 5 h. Most suitable for use as probiotic strains *B. clausii* BA8 and *B. subtilis* BA11, sequenced via Illumina NovaSeq, showed average nucleotide identity (ANI) values of 98.1% and 97.8%, respectively. The genome annotation of *B. clausii* and *B. subtilis* with Prokka revealed 4,498,248‐4,215,606 bp genome length, 44%–43% GC content, and 110–26 contigs, respectively. *B. clausii* BA8 has been comprehensively characterized, is of low risk for human consumption, and has been recommended as a potential probiotic strain. However, further in vivo experimentation is required to confirm these findings.

## Graphical abstract

*Bacillus clausii* BA8 and *Bacillus subtilis* BA11 strains can be used as alternatives to LABs. Identifying key genetic features of *B. subtilis* BA8 and *B. clausii* BA11 strains with whole genome sequencing. The strains robust resistance to harsh gastrointestinal tract (GIT) conditions and strong aggregation abilities.

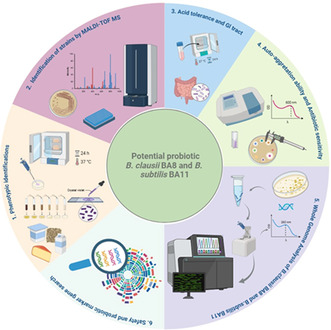

## 1 Introduction

The *Bacillus* genus comprises aerobic, endospore‐forming, Gram‐positive bacteria capable of both heterotrophic and autotrophic growth, utilizing diverse carbon sources. This genus encompasses more than 200 new species, with certain classifications refined based on new biological data (Kahraman et al. 2024). *Bacillus* species are generally considered soil microorganisms but can be isolated from silage, milk, and cheese (Ertürkmen and Öner 2023). After entering the gastrointestinal (GI) tract, *Bacillus* spores germinate into metabolically active vegetative cells and elicit their probiotic effect (Susanti et al. 2021). They may be undiscovered GI tract commensal and probiotics that can survive in any ecosystem longer than vegetative forms of microorganisms (Ahire, Kashikar, and Madempudi 2021). The outstanding resistance and dormancy of these endospores mainly help the bacterial cells survive in the absence of water/nutrients or harsh conditions such as extreme temperature, desiccation, and acidic pH (Ghelardi et al. 2022; Todorov et al. 2022; Khokhlova et al. 2023). These properties make them advantageous against probiotic species such as *Lactobacillus* and *Bifidobacteria* (Bhushan et al. 2019; Ahire, Kashikar, and Madempudi 2021; Alp 2022; Ertürkmen et al. 2024). Studies have proven that *Bacillus* strains can suppress undesirable microorganisms by producing various enzymes, antimicrobial peptides, and metabolites, thus supporting the intestinal microbiota (Grant, Gay, and Lillehoj 2018). In recent years, foods and feeds containing probiotic *Bacillus* are generally used as nutritional supplements for humans, animal growth promoters, and aquaculture growth regulators or disease resistance providers (Lee, Kim, and Paik 2019).

In studies where resistance and adaptation to conditions within the gastrointestinal tract are successful, it is stated that endospore‐forming bacteria, such as *Bacillus* adhere to the gut and exhibit species‐specific survivor ability (Todorov et al. 2022). Different and commonly used probiotics belonging to *Bacillus* include *B. subtilis*, *B. coagulans*, *B. indicus*, *B. licheniformis*, *B. clausii*, *B. amyloliquefaciens* and *B. licheniformis* (Lee, Kim, and Paik 2019; Todorov et al. 2022). Researchers have indicated that *B. subtilis, B. clausii* (now *Shouchella clausii*), *B. velezensis*, and *B. pumilus* strains have many probiotic properties such as antagonistic activity, an adhesion/aggregation ability, gastric enzyme, and Ox‐Bile resistance (Ramlucken et al. 2020; Sam‐on et al. 2022). The ability of probiotic strains to adhere to host epithelium can be attributed to cell surface proteins (Alp 2022). *Bacillus* strains have also reported biofilm formation, which is associated with adhesion and show good adhesion ability (Peng et al. 2020; Louis et al. 2022). Among these strains, the most preferred strain for use as a probiotic culture in the past and today is *B. clausii* (Saroj, Ahire, and Shukla 2023).

Strains that can be used as probiotics should not cause problems with antibiotic resistance and should not carry transferable antibiotic resistance genes (ARG) (Imperial and Ibana 2016). Concerns may arise regarding the safety of *Bacillus* species due to their potential to produce toxins and biogenic amines and their capability for ARG transfer. Due to these concerns, whole‐genome (WG) analysis has become a prominent method for accurately assessing these species' risks to human health and for performing safety evaluations (Fraccalvieri et al. 2022). Aminoglycoside antibiotics are potent antibiotics with bacteriostatic and bactericidal effects used to treat bacterial infections (Krause et al. 2016). Studies in *Bacillus* spp. that the *B. clausii* , is erythromycin‐resistant, indicated to be associated with the *erm* (Bozdogan, Galopin, and Leclercq 2004) and *aadD2* (Bozdogan et al. 2003) genes, which are not transferable through conjugation. Macrolide resistance conferred by the *erm* gene allows the probiotic to persist in the intestine when administered alongside oral macrolides (Bozdogan, Galopin, and Leclercq 2004).

The survivability of *Bacillus* strains in the GI tract and their thermal stability makes them particularly appealing for probiotic evaluation, as their resilience in challenging environments has increased utilization. This study aimed first to identify potential probiotic *Bacillus* spp. strains at the species level using MALDI‐TOF MS. Additionally, it assessed the strains' GI tract survival, auto‐aggregation abilities, and phenotypic antibiotic resistance characteristics. Genome sequencing of *B. clausii* and *B. subtilis* strains, which demonstrated the highest survival rates and auto‐aggregation ability in the GI tract environment, was conducted on the Illumina NovaSeq platform. This approach facilitated a thorough classification of *Bacillus* species and the prediction of associated genes, with a focus on safety and probiotic properties, thereby providing valuable insights into the probiotic potential of these strains.

## 2 Material and Methods

### 2.1 Phenotypic Identifications and Biogenic Amine Forming Capabilities of Potential Probiotic Strains

Potential probiotic strains isolated from different types of cheese, silage, and milk samples from Turkey. In the first stage, various characteristics of the strains were determined, such as Gram‐endospore staining, catalase, indole, citrate, urea, Voges‐Proskauer (VP), and triple sugar iron (TSI) test for phenotypic identification (Loeza‐Lara et al. 2005). To evaluate the capacity for producing biogenic amines, overnight cultures were inoculated (1%) into the Trypto‐Casein Soy (TCS) (Biokar diagnosis, France) agar enriched with a 2% variety of amino acids (histidine, tyrosine, lysine, ornithine, phenylalanine, and tryptophan) at 37°C for 24 h, following the protocol described by Joosten and Northolt (1989).

### 2.2 Identification of Strains by MALDI‐TOF MS

Strains were identified by MALDI‐TOF MS Autoflex Speed (Bruker Daltonics GmbH, Germany) using the extended direct transfer method instructed by the manufacturer. Calibration of the method (MBT_FC.par) was performed using BTS (Bacterial testing standard, Bruker, Germany), which contains eight different MS (mass spectra) peaks ranging from 2000 to 20,000 Da m/z. The matrix used for microorganism identification was α CHCA (10 mg/mL), and samples were detected on the MTP 384 Ground Steel Target. MS signals for each sample were acquired in linear positive mode, and laser intensity was used between 55% and 60%. Mass spectra were processed using Biotyper 3.4 software (Bruker Daltonics GmbH, Germany). The match between the spectra obtained from the microorganism isolates and the spectrum of the reference profile was expressed according to a score made by the Biotyper 3.4 software. This score value was also visualized as a color scale (colors corresponding to the score value; > 1.99 green; > 1.69 yellow; 0.0–1.69 red) (Lartigue 2013; Karasu‐Yalçın et al. 2021).

### 2.3 Acid Tolerance and Gastrointestinal Tract Enzyme Test

The *Bacillus* strains are cultivated in TCS broth (Biokar Diagnosis, France) for 18 h at 37°C. Fresh cultures were inoculated (at a concentration of 1%) into 10 mL of phosphate‐buffered saline solution (PBS), with a pH adjusted to 2.0 and 3.0, respectively. Following this procedure, the cultures were incubated at 37°C for 3 h. *Lactiplantibacillus* strains were cultured in De Man, Rogosa, and Sharpe broth (MRS) (Merck, Germany) for 18 h at 37°C. The process was continued using the same methodology for these strains. Samples were taken from the cultures at 0, 1, and 3 h, and viability was enumerated using the serial plating method on TCS and MRS agar (Alp and Kuleaşan 2020). Formula (1) described by Tokatlı et al. (2015) was used to calculate the survival percentage of cultures.

(1) $$\%\text{survival}=\frac{\log\;\mathrm{cfu}\;\text{of initial viable surviving cell}}{\log\;\mathrm{cfu}\;\text{of initial viable inoculated cells}}\times 100$$

To evaluate the resistance of the strains to the pepsin enzyme, a solution of PBS was adjusted to pH 2.0 and 3.0, with the addition of 3 mg/mL pepsin enzyme (Sigma‐Aldrich, Germany). This procedure was conducted using Horácková, Žaludová, and Plocková (2011) methodology. In vitro determination of resistance to pancreatin and Ox‐Bile was conducted by adding 1% Ox‐Bile (Merck, Germany) and 1 mg/mL pancreatin (Sigma‐Aldrich, Germany) to PBS adjusted to pH 8.0. Subsequently, fresh cultures were inoculated into the freshly prepared solutions at a concentration of 1% and incubated at 37°C for 4 h. To determine cell viability, samples were taken at 0. and 4 h, and serial dilutions were plated on TCS and MRS agar (Sahadeva et al. 2011; Ahire, Kashikar, and Madempudi 2021). The tolerance of the strains used in the study to phenol was determined by preparing two groups of TCS broth media, 0.3% (v/v) containing and without phenol, and inoculating fresh cultures (1%) (Kılıç et al. 2013). Samples were taken from the fresh cultures at the beginning and 24th hour of incubation. Enumeration was done by plating on TCS agar to determine the viability of the cells. The process was performed in an MRS medium to determine phenol resistance in the *Lactiplantibacillus* strain.

### 2.4 Auto‐Aggregation Ability Assays

Determined to auto‐aggregation abilities of *Bacillus* strains using the method described by Kos et al. (2003). The *Bacillus* strains were grown in TCS broth, and the *Lactiplantibacillus* strain was grown in MRS broth. The strains were incubated at 37°C for 18 h, and at the end of this process, they were centrifuged at 4000 rpm for 15 min. Apart from this, the optical density of the cultures was set to 0.6 absorbance at 600 nm in the spectrophotometer (Optizen Pop Nano Bio, Mecasys Co. Ltd., Korea). The tested strains were incubated for 5 h for the auto‐aggregation assay, and the aggregation rate was calculated using formula (2), as described by Collado and Sanz (2006).

(2) $$\%\text{Autoaggregation}=\left(1-\left[A_{t}/A_{0}\right]\right)\times 100$$

(A_0_ is the first optical density, and A_t_ is the optical density after 5 h).

### 2.5 Assessment of Antibiotic Sensitivity

The antimicrobial susceptibility of the strains was determined using the Kirby‐Bauer disc diffusion method, following the standard protocol outlined by the European Committee on Antimicrobial Susceptibility Testing (EUCAST, 2017). Twelve antibiotic discs (Oxoid, England), including Penicillin G (10 U), Ampicillin (10 μg), Novobiocin (30 μg), Erythromycin (15 μg), Tetracycline (30 μg), Gentamicin (10 μg), Oxytetracycline (30 μg), Rifampicin (5 μg), Nitrofurantoin (300 μg), Ciprofloxacin (5 μg), Chloramphenicol (30 μg), Oleandomycin (15 μg) were used. The diameter of the inhibition zones surrounding the antibiotic discs was measured, and all strains were classified as susceptible (S), intermediate‐resistant (I), or resistant (R) based on EUCAST criteria for *Bacillus* species. In addition, a modification (instead of Muller Hinton agar) was made to the methodology employed for the antibiotic susceptibility test, whereby TCS agar.

### 2.6 Whole Genome Sequencing of Potential Probiotic *B. clausii* BA8 and *B. subtilis* BA11

A commercial company (BM Labosis, BM Lab. Ltd. Sti. Ankara, Turkey) conducted strain identification and analysis of ARG. Two main methods were used for quality control of DNA samples during library preparation, construction, and analysis: 1. The size distribution and degree of DNA degradation were monitored on 1% agarose gels. 2. DNA purity and concentration were checked using the NanoPhotometer spectrophotometer (IMPLEN, USA). Accurate quantification of DNA was measured using the Qubit DNA Assay Kit in the Qubit 2.0 Fluorometer (Life Technologies, USA). A 1.0 μg of genomic DNA (gDNA) per sample was utilized for DNA sample preparations. Sequencing libraries were prepared using the NEBNext DNA Library Prep Kit. The gDNA was randomly fragmented to a size of 350 bp, end‐polished, A‐tailed, and ligated with the NEBNext adapter for Illumina sequencing. PCR products were purified, and resulting libraries were analyzed for size distribution using the Agilent 2100 Bioanalyzer. The Illumina NovaSeq 6000 (Illumina Inc., San Diego, CA, USA) was employed as the sequencing platform to generate 1 GB of data per sample using the 2 × 150 paired‐end sequencing method. Raw data quality was controlled with FastQC, and low‐quality reads were filtered out with trimmomatic. Bowtie2 performed mapping to the reference genome, and variant calling was done with bcftools. The average nucleotide identity (ANI) of *B. clausii* BA8 and *B. subtilis* BA11 was analyzed using the EZBioCloud ANI Calculator (https://www.ezbiocloud.net/tools/ani). Genomeannotation of the strains predicted using Prokka, a tool that identifies coding sequences, rRNAs, tRNAs, and other genomic features, facilitating downstream functional analysis. The quality of the genome assemblies was evaluated using QUAST (Quality Assessment Tool for Genome Assemblies). Variant annotation was carried out using snpEff, and final contigs were generated with vcf‐consensus. ARG in *B. clausii* and *B. subtilis* were identified using ABRicate (https: //github.com/tseemann/abricate) (Jia et al. 2017; Sam‐on et al. 2023).

### 2.7 Statistical Analysis

Microbial counts of the study's acid tolerance and GI tract enzyme analyses were performed in triplicate. Results are presented as mean ± standard deviation. Statistical software Minitab 17 (Minitab Inc., State College, PA, USA) was used for data analysis. One‐way ANOVA determined differences at a significance level of *p* < 0.05.

## 3 Results and Discussion

### 3.1 Characterization and Identification of Strains

**FIGURE 1:**
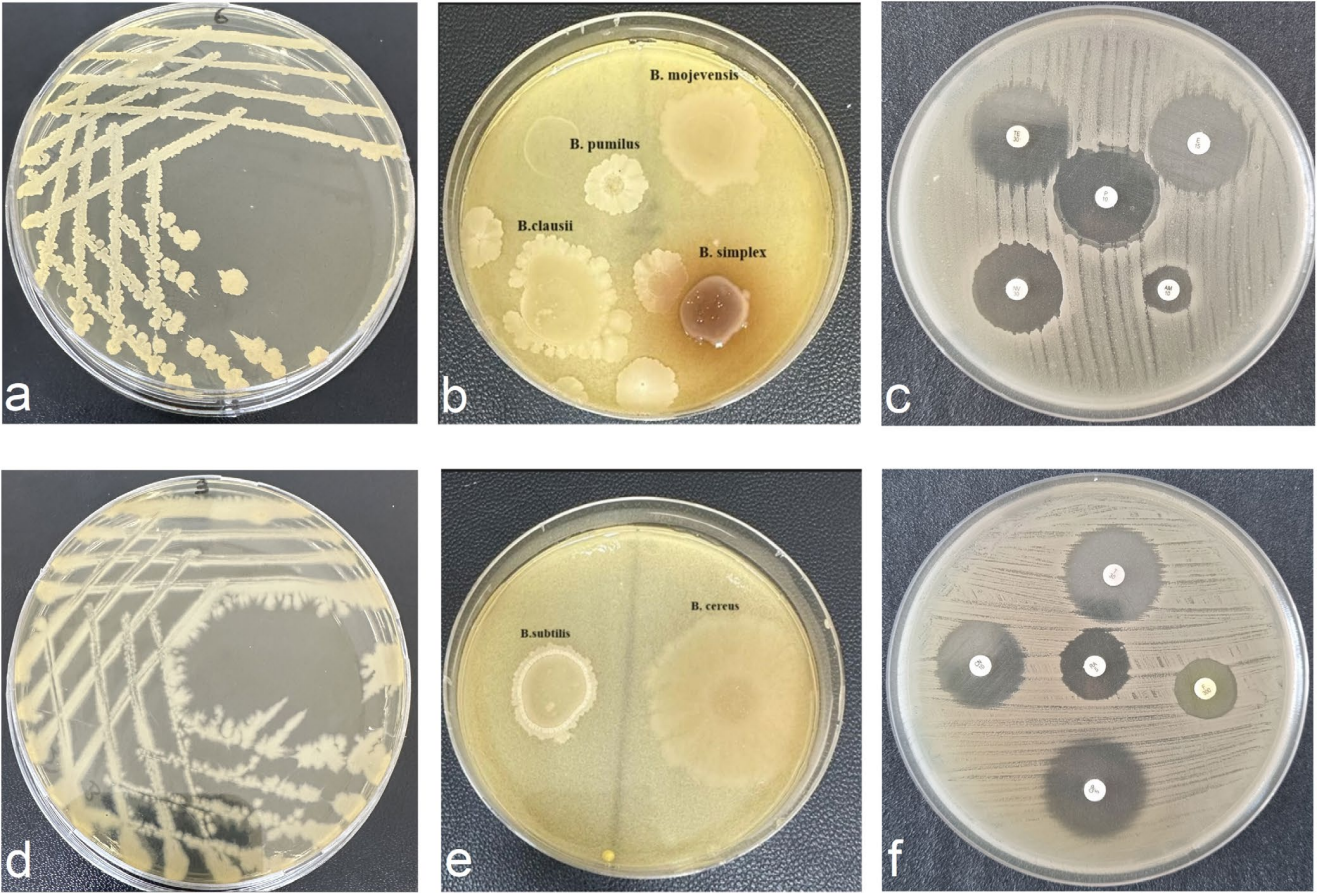
Colony images of the pure strains of *B. clausii* BA8 (a) and *B. subtilis* BA11(d); For possible biogenic amine forming capacities a positive reaction with a red‐brown zone for *B. simplex* (b) and characterized negative reactions with transparent zones for *B. subtilis* and *B. cereus* (e) in the 2% tyrosine amino acid in TCS medium; Antibiotic disc zones of *B. clausii* BA8 (c) and *B. subtilis* BA11 (f).

**FIGURE 2:**
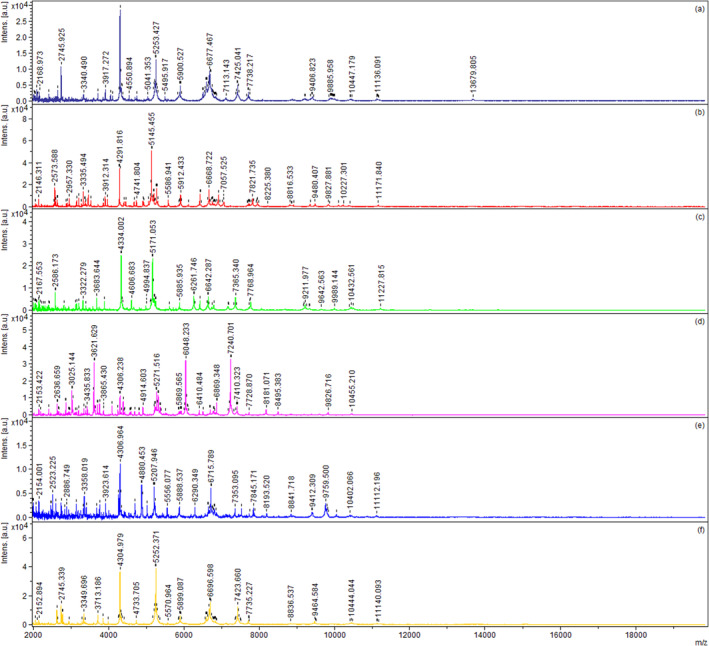
, *clausii* *simplex* MALDI‐TOF mass spectra some potential probiotic Bacillus species. (a) B.*mojavensis*, (b) B. *clausii*, (c) B. *cereus*, (d) B. *pumilus,* (e) B. *simplex*, (f) B. *subtilis*, respectively.

This study obtained 17 bacterial isolates, including 14 *Bacillus* spp. and 3 *Lactiplantibacillus* from traditional cheese, milk, and silages in Turkey. The isolates were subjected to morphological, microscopic, and biochemical analyses. As shown in Table 1, all isolates were determined to be Gram‐positive. Endospore staining revealed that isolates BA15, BA16, and BA17 were non‐spore‐forming, while the remaining *Bacillus*‐like isolates exhibited central spore formation. Catalase tests were positive, and indole and urea tests yielded negative results across these isolates. The isolates also tested positive or weak for citrate utilization and VP reaction. Various phenotypic characteristics of the strains were determined, and it was observed that most of them did not decarboxylate ornithine, histidine, tyrosine, tryptophane, phenylalanine, or lysine amino acids. Two strains produced a red‐brown zone from tyrosine (Figure 1). The strains were identified using MALDI‐TOF MS; 10 strains received a score of ≥ 2.0 at the species level, while seven strains received a score between 1.8 and 2.0 at the genus level (Table 1). Seventeen isolates of bacteria were identified as 3 *Lpb. plantarum*, 5 *Paenibacillus*, 9 *Bacillus* spp. (*B. mojevensis*, *B. simlex*, *B. clausii* , *B. cereus*, *B.subtilis*, *B. pumilis*). Researchers have reported commonly isolating *Clostridium* and *Bacillus* (Ertürkmen and Öner 2023) as well as *Paenibacillus spp*. (Driehuis, Hoolwerf, and Rademaker 2016) from silage, raw milk, and cheese samples obtained from dairies. Additionally, it has been noted that endospore‐forming bacteria are particularly dominant in corn silage (Borreani et al. 2019). The comparison of MALDI‐TOF mass spectra, which demonstrates interspecies identification differences of some potential probiotic *Bacillus* strains obtained from this study, is presented in Figure 2.

**TABLE 1:** Preliminary characteristics of strains.

Strain code	Source	Morphology			MALDI‐TOF MS		Phenotypic descriptions				Biogenic amine‐forming capabilities					
		Microscopic image	Gram reaction	Catalase	Identification name	Score value	İndole	Citrate	Urea	VogesProskauer (VP)	Orn	His	Tyr	Trp	Phe	Lys
BA1	Silage	B	+	+	*P. polymyxa*	1.815 ± 0.12	−	+	−	+	−	0	−	−	−	−
BA2	Cheese	B	+	+	*P.illinoisensis*	1.913 ± 0.34	−	+	w	+	−	−	−	−	−	−
BA3	Cheese	B	+	+	*B. mojevensis*	2.285 ± 0.09	−	w	−	+	−	−	−	−	−	−
BA4	Silage	B	+	+	*P. macarans*	1.913 ± 0.15	−	w	−	+	−	−	−	−	−	−
BA5	Cheese	B	+	+	*B. simlex*	2.014 ± 0.09	−	+	−	+	−	−	+^a^	+^a^	−	−
BA6	Cheese	B	+	+	*P. amylolyticus*	1.951 ± 0.16	−	+	−	+	−	−	−	−	−	−
BA7	Cheese	B	+	+	*P. polymyxa*	1.837 ± 0.38	−	+	−	+	−	−	−	−	−	−
BA8	Cheese	B	+	+	*B. clausii*	2.152 ± 0.11	−	+	−	+	−	−	−	−	−	−
BA9	Milk	B	+	+	*B.cereus*	1.902 ± 0.32	−	+	w	+	−	−	−	+	−	−
BA10	Cheese	B	+	+	*B. pumilis*	2.102 ± 0.22	−	+	−	−	−	−	−	−	−	−
BA11	Cheese	B	+	+	*B. subtilis*	2.228 ± 0.08	−	+	−	+	−	−	−	−	−	−
BA12	Milk	B	+	+	*B. pumilus*	1.834 ± 0.14	−	w	w	−	−	−	+	−	−	−
BA13	Cheese	B	+	+	*B. cereus*	2.283 ± 0.07	−	+	−	+	−	−	+	−	−	−
BA14	Milk	B	+	+	*B. subtilis*	1.934 ± 0.22	−	+	−	+	−	−	−	−	−	−
BA15	Cheese	Lpb	+	−	*Lpb. plantarum*	2.225 ± 0.09	−	−	−	−	−	−	−	−	−	−
BA16	Cheese	Lpb	+	−	*Lpb. plantarum*	1.941 ± 0.07	−	w	−	−	−	−	−	+	−	−
BA17	Cheese	Lpb	+	−	*Lpb. plantarum*	1.996 ± 0.11	−	−	−	−	−	−	−	−	−	−

Abbreviations: −: Negative reaction; +: Positive reaction, B: *Bacillus*, His: Histidine, Lpb: *Lactiplantibacillus*, Lys: Lysine, Orn: Ornithine, Phe: Phenilalanine, Trp: Tyrptophane, Tyr: Tyrosine, W: Weak reaction.

^a^ Reaction (red‐brown zone).

### 3.2 Probiotic Properties of Strains

**FIGURE 3:**
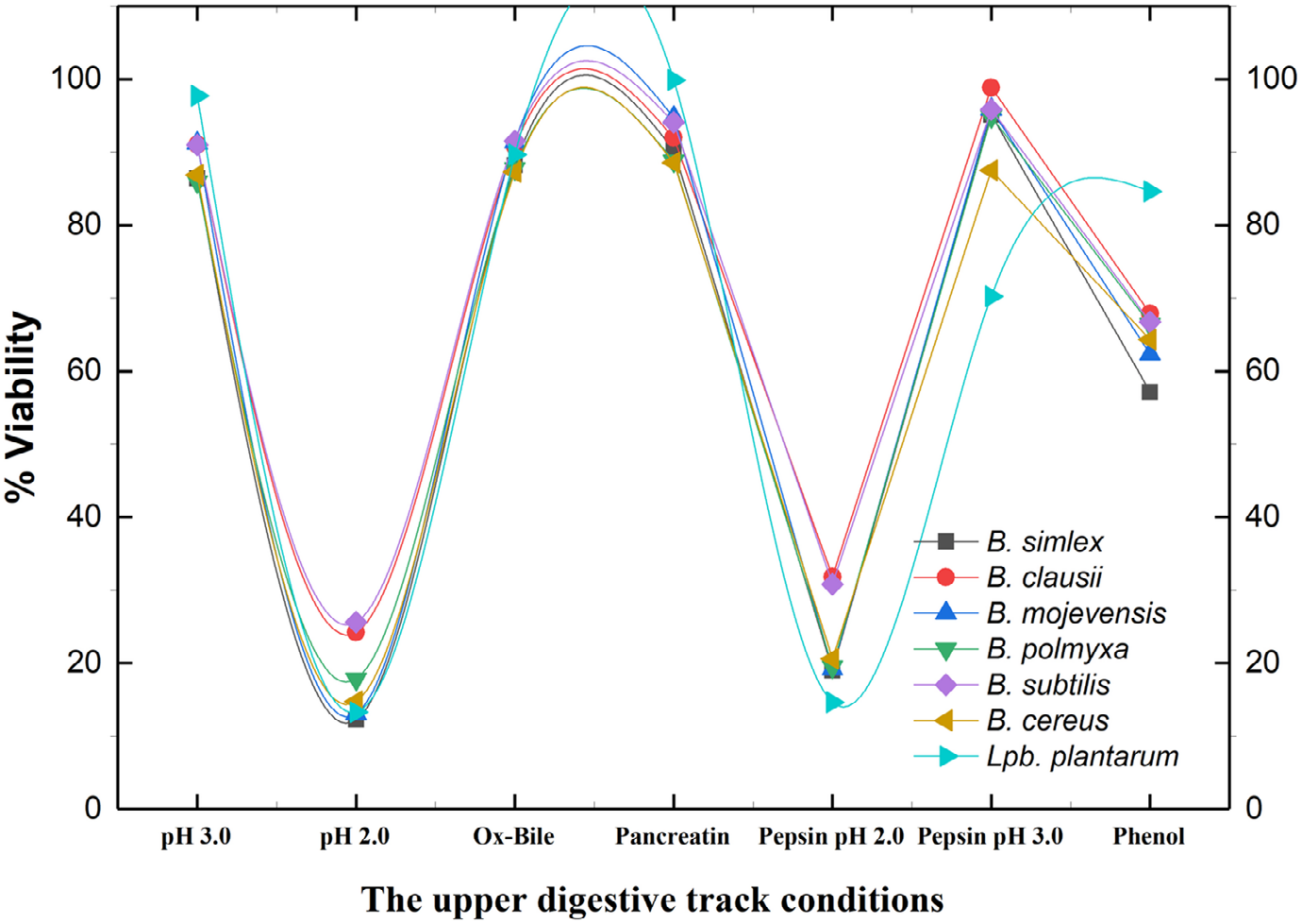
The viability rates of the strains when confronted with upper digestive system conditions, including low pH (2.0 and 3.0), pepsin‐pancreatin enzymes, and Ox‐Bile.

The strains of 6 *Bacillus* spp. and 1 *Lpb. plantarum* strain (widely used as a probiotic) with a MALDI‐TOF score of ≥ 2.0 and good safety characteristics, various probiotic properties were investigated. The results of the loss of viability of the strains are shown in Table 2, and Figure 3 gives the viability rates of the strains against upper digestive tract conditions. It has been reported that the resistance of each bacterial species to the proteolytic effect is related to the amino acid content of membrane proteins, also the acid tolerance response may vary from species to species and this resistance ability may depend on the concentration of hydronium ions accumulated in the cell (Escobar‐Ramírez et al. 2020). However, most bacteria pass through this pH level by being protected within food matrices (Todorov et al. 2022). The present study aimed to determine the resistance properties of the strains to the pepsin enzyme at pH 2.0 and 3.0. The findings indicated that the strains exhibited high resistance at pH 3.0. While *Bacillus* strains demonstrated minimal resistance at pH 2.0, this resistance was significantly enhanced at pH 3.0. The viability of the *Bacillus* strains was found to be in the range of 87.49%–98.86% at pH 3.0. *B. clausii* BA8 strain, which stands out with many features, showed low viability (31.84%) at pH 2.0 but showed a decrease of less than a logarithm at pH 3.0 in both the initial and final stages of the analysis, and its final viability was 98.86%. When the results of *Lpb. plantarum* LA15 were compared with the results of the *Bacillus* strains in the current work, it was seen that they were superior to each other in different conditions. *Lpb. plantarum* LA15 showed the highest viability percentage (97.73%) after 3 h at pH 3.0. It was followed by *B. clausii* BA8, *B. mojevensis* BA3, *B. subtilis* BA11.

**TABLE 2:** The viability results of microorganisms at pH 3.0, pH 2.0 and against 3 mg/mL pepsin, 1 mg/mL pancreatin and %1 Ox‐Bile (log CFU/mL).

Microorganism	Viability counts at pH 2.0		% Viability
	0 h	3 h	0–3 h
*B. simlex* BA5	8.17 ± 1.09^ab^	1.00 ± 0.00^d^	12.23 ± 0.07
*B. clausii* BA8	8.30 ± 0.28^a^	2.00 ± 0.01^a^	24.22 ± 1.40
*B. mojevensis* BA3	8.25 ± 0.28^a^	1.08 ± 0.00^c^	13.09 ± 0.76
*B. pumilus* BA10	8.42 ± 1.16^a^	1.50 ± 0.00^b^	17.83 ± 1.06
*B. subtilis* BA11	7.83 ± 0.66^ab^	2.00 ± 0.01^a^	25.60 ± 1.42
*B. cereus* BA13	7.38 ± 0.27^b^	1.09 ± 0.01^c^	14.75 ± 0.39
*Lpb. plantarum* LA15	8.13 ± 0.01	1.08 ± 0.00	13.28 ± 0.01

	Viability counts at pH 3.0% viability		% Viability
	0 h	3 h	0–3 h
*B. simlex* BA5	8.20 ± 1.05^a^	7.09 ± 1.00^a^	86.41 ± 0.07
*B. clausii* BA8	8.42 ± 1.28^a^	7.66 ± 0.49^a^	91.00 ± 1.12
*B. mojevensis* BA3	8.32 ± 0.03^a^	7.59 ± 0.47^a^	91.24 ± 0.62
*B. pumilus* BA10	8.49 ± 1.16^a^	7.29 ± 1.40^a^	85.94 ± 0.34
*B. subtilis* BA11	8.12 ± 1.16^a^	7.39 ± 0.53^a^	91.01 ± 0.88
*B. cereus* BA13	8.15 ± 1.27^a^	7.08 ± 0.87^a^	86.88 ± 0.57
*Lpb. plantarum* LA15	7.93 ± 0.05	7.75 ± 0.13	97.73 ± 2.06

	Viability counts at pepsine enzyme at pH 2.0		% Viability
	0 h	3 h	0–3 h
*B. simlex* BA5	7.14 ± 0.13^a^	1.29 ± 0.06^b^	18.09 ± 0.04
*B. clausii* BA8	7.20 ± 0.12^a^	2.29 ± 0.05^a^	31.84 ± 0.04
*B. mojevensis* BA3	6.99 ± 0.76^a^	1.34 ± 0.19^b^	19.21 ± 0.41
*B. pumilus* BA10	6.31 ± 0.75^ab^	1.23 ± 0.10^b^	19.48 ± 0.19
*B. subtilis* BA11	7.19 ± 0.13^a^	2.21 ± 0.27^a^	30.81 ± 0.09
*B. cereus* BA13	5.69 ± 0.51^b^	1.17 ± 0.14^b^	20.58 ± 0.27
*Lpb. plantarum* LA15	8.13 ± 0.76	1.19 ± 0.02	14.64 ± 0.02

	Viability counts at pepsine enzyme at pH 3.0		% Viability
	0 h	3 h	0–3 h
*B. simlex* BA5	7.39 ± 0.14^a^	7.03 ± 0.76^a^	95.13 ± 0.44
*B. clausii* BA8	7.62 ± 0.06^a^	7.54 ± 0.48a	98.86 ± 0.30
*B. mojevensis* BA3	7.25 ± 0.16^a^	6.95 ± 0.56^a^	95.86 ± 0.28
*B. pumilus* BA10	6.98 ± 0.24^ab^	6.62 ± 0.97^ab^	94.90 ± 0.19
*B. subtilis* BA11	7.70 ± 0.73^a^	7.37 ± 0.13^a^	95.81 ± 0.42
*B. cereus* BA13	6.28 ± 0.18^b^	5.49 ± 0.38^b^	87.49 ± 0.13
*Lpb. plantarum* LA15	8.17 ± 0.09	5.74 ± 0.12	70.25 ± 1.33

	Viability counts at Ox‐Bile		% Viability
	0 h	4 h	0–4 h
*B. simlex* BA5	7.69 ± 0.18^ab^	6.78 ± 0.40^a^	88.22 ± 0.15
*B. clausii* BA8	7.85 ± 0.06^a^	7.13 ± 0.69^a^	90.77 ± 0.45
*B. mojevensis* BA3	7.83 ± 0.48^a^	7.15 ± 0.58^a^	91.41 ± 0.07
*B. pumilus* BA10	6.89 ± 0.12^c^	6.04 ± 0.38^a^	87.73 ± 0.19
*B. subtilis* BA11	7.06 ± 1.26^bc^	6.46 ± 2.49^a^	91.54 ± 0.87
*B. cereus* BA13	6.94 ± 0.55^c^	6.05 ± 1.00^a^	87.30 ± 0.32
*Lpb. plantarum* LA15	8.43 ± 0.06	7.56 ± 0.12	89.67 ± 2.01

	Viability counts at pancreatin enzyme		% Viability
	0 h	4 h	0–4 h
*B. simlex* BA5	8.43 ± 0.15^a^	7.61 ± 0.25^a^	90.32 ± 0.07
*B. clausii* BA8	8.37 ± 0.72^a^	7.70 ± 0.19^a^	91.99 ± 0.38
*B. mojevensis* BA3	8.19 ± 0.48^a^	7.76 ± 0.24^a^	94.75 ± 0.17
*B. pumilus* BA10	8.11 ± 0.48^a^	7.20 ± 0.65^a^	88.82 ± 0.19
*B. subtilis* BA11	7.95 ± 0.56^a^	7.48 ± 0.32^a^	94.07 ± 0.17
*B. cereus* BA13	8.08 ± 0.67^a^	7.16 ± 0.48^a^	88.56 ± 0.14
*Lpb. plantarum* LA15	8.39 ± 0.30	8.38 ± 0.32	99.88 ± 1.06

	Viability counts at phenol		% Viability
	0 h	24 h	0–24 h
*B. simlex* BA5	7.52 ± 0.15^a^	4.29 ± 0.09^a^	57.15 ± 0.04
*B. clausii* BA8	7.78 ± 0.27^a^	5.28 ± 0.30^a^	67.86 ± 0.02
*B. mojevensis* BA3	7.23 ± 0.10^a^	4.51 ± 0.81^a^	62.33 ± 0.50
*B. pumilus* BA10	6.54 ± 0.34^b^	4.34 ± 0.22^a^	66.43 ± 0.08
*B. subtilis* BA11	7.51 ± 0.50^a^	5.01 ± 0.16^a^	66.75 ± 0.47
*B. cereus* BA13	6.50 ± 0.51^b^	4.18 ± 1.08^a^	64.32 ± 0.40
*Lpb. plantarum* LA15	8.00 ± 0.10	6.77 ± 0.08	84.62 ± 0.08

*Note:* The letters 'a, b' indicate significant differences (*p* < 0.05).

When pepsin enzyme was added to a low pH medium, the strains showed approximately 7 logarithmic reductions in cell viability, especially at pH 2.0, and approximately 3 logarithmic reductions at pH 3.0 (*p* < 0.05). A similar situation applies to the results against the pancreatin enzyme. Pancreatin is secreted immediately after the stomach in humans, resulting in a notable change in pH from 3.0 to 8.0. The isolates were resistant to pancreatin, with minimum and maximum survival rates determined as 88.56% and 94.75%, respectively. *Lpb. plantarum* LA15 exhibits a markedly high survival rate against both the pancreatin enzyme and Ox‐Bile, with values of 99.88% and 89.67%, respectively.

Ox‐Bile has been identified as a significant factor influencing the survival of probiotic strains within the GI tract. In experiments simulating the human GI tract, the Ox‐Bile concentration is typically within the range of 0.3% to 0.5% by volume per weight (v/w). However, in this study, we tested their viability against 1% (v/w). Despite this, the strains showed a suitable survival rate against Ox‐Bile. The highest viability belongs to *B. subtilis* BA11 (91.54%). It is followed by *B. mojevensis* BA3 and *B. clausii* BA8. The *B. clausii* BA8 strain used in the present study exhibited 91.00% survivability at 3 h of incubation at pH 3.0 and 90.77% survivability at Ox‐Bile. These findings are higher than those reported by Patel, Patel, and Acharya (2020) in their studies with *B. clausii* . Researchers have stated that bacterial resistance to high concentrations of Ox‐Bile may stem from a stress adaptation mechanism developed through prior exposure to analogous conditions (Sahadeva et al. 2011). In the current work, this may be associated with the natural adaptation of the strains, especially in the Ox‐Bile resistance results, given that they were isolated from a range of cheeses with high salt content.

When the results of phenol resistance are examined, it is seen that the highest resistance belongs to *Lpb. plantarum* LA15 (84.62%). *B. clausii* BA8 follows it, there was a 2.5 logarithmic decrease in cell count compared to initially, and it showed 67.86% viability. Other strains *B. pumilus* BA10 and *B. subtilis* BA11 showed also good resistance against phenol; their survival was 66.43% and 66.75%, respectively. Phenol, a toxic metabolite that is secreted as a result of the deamination of some amino acids and can move through the colon, is expected to be tolerated in the body with the help of potential probiotics (de Oliveira et al. 2017; Fonseca et al. 2021). For this purpose, the present study investigated whether the isolates were resistant to phenol. The highest resistance belongs to *Lpb. plantarum* LA15 (84.62%), it is followed by *B. clausii* BA8 (67.86%). Other strains also showed good resistance against phenol; their survivability was above 65% average. Although tested *Bacillus* spp. survival percentage to phenol is low compared to studies conducted with LABs, it is good considering that this is the first study to examine this feature of potential *Bacillus* strains, and the viability of the strains is over 65%.

### 3.3 Auto‐Aggregation

**FIGURE 4:**
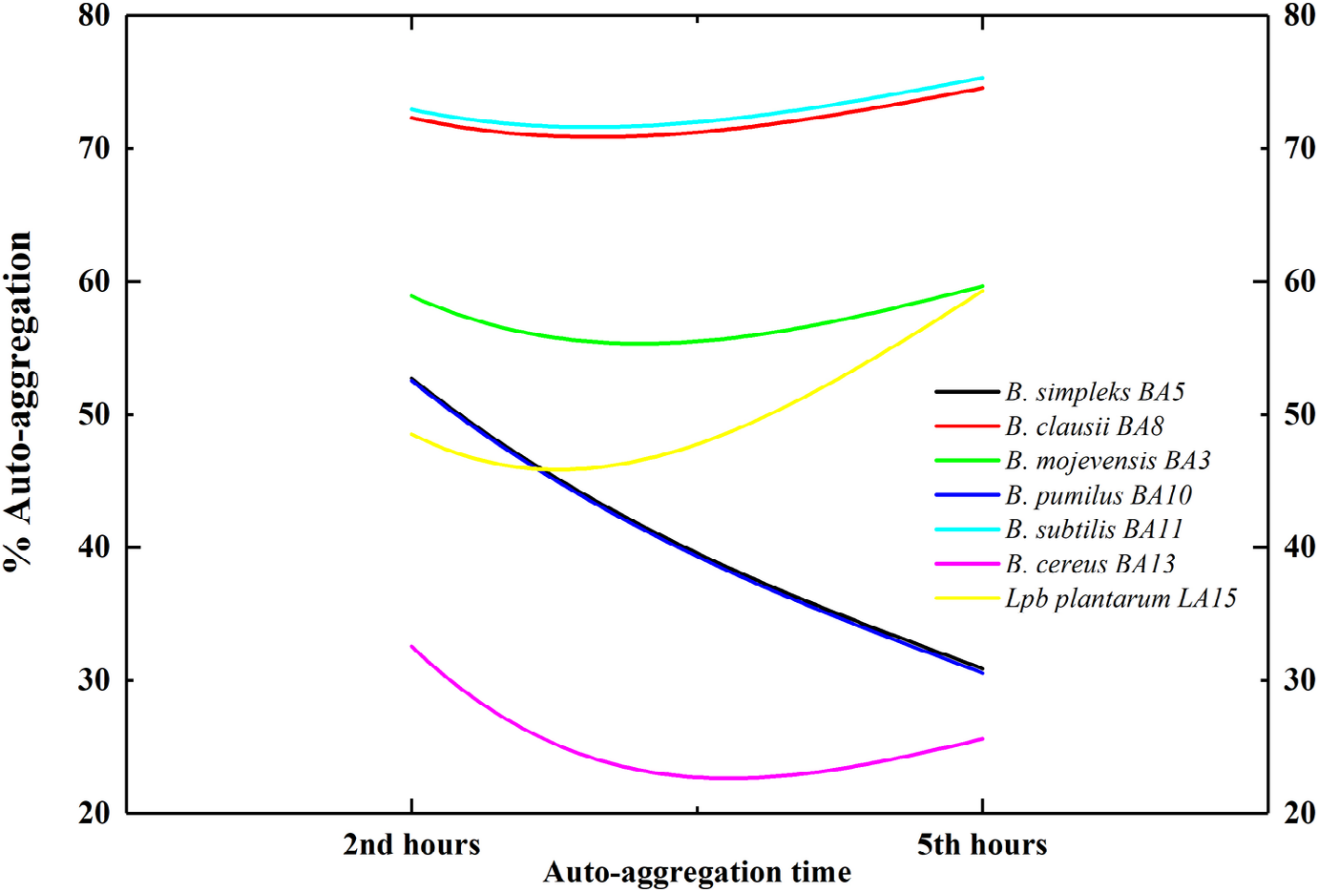
The results of the percentage auto‐aggregation (a process that protects against environmental stresses and host responses) of all the strains that were tested at the end of 2 and 5 h.

The strains in this study demonstrated auto‐aggregation ability at varying degrees (Figure 4). The auto‐aggregation abilities of the isolates ranged from 30.92% to 75.32% after 5 h of incubation. Although all strains retained their auto‐aggregation ability after 2 and 5 h, a significant decrease in the aggregation ability of *B. cereus* BA13 was observed. At the end of 5 h, *B. subtilis* BA11 and *B. clausii* BA8 showed the highest auto‐aggregation ability at 75.32% and 74.55%, respectively. In the study, these two strains showed higher auto‐aggregation ability at the end of 5 h compared to *Lpb. plantarum* LA15 (59.31%). The researchers indicated that lipoteichoic acid (a protein present on the surface of cells), carbohydrates, and other factors influence cell aggregation. This property is crucial in determining whether cells adhere to the gut and preventing pathogenic microorganisms' colonization (Rodríguez‐Sánchez et al. 2021). In addition, studies have stated that the reason auto‐aggregation levels are detected at different levels may depend on the strain or may be due to the physicochemical properties of the cell surface (Alp and Kuleaşan 2020; Alp 2022). In the present study, the two strains with the highest auto‐aggregation ability were *B. clausii* BA8 (74.55%) and *B. subtilis* BA11 (75.32%), as determined by analysis of all strains tested. The auto‐aggregation abilities of *B. simplex* BA5 and *B. pumilus* BA10 exhibited a notable decline after the fifth hour, with the auto‐aggregation results determined to be 30.92% and 30.60%, respectively.

### 3.4 Assessment of Antibiotic Sensitivity

Antibiotic sensitivity tests were performed for 7 isolates using 12 different antibiotic disks using the disk diffusion method (Table 3). Three of the tested strains (*B. mojevensis* BA3, *B. pumilus* BA10 and *Lpb. plantarum* LA15) were found to be sensitive to Penicillin G, which belongs to the β‐lactamase group, while the other four strains were found to be resistant. To Ampicillin, another antibiotic belonging to this group, *B. pumilus* BA10 was sensitive, *Lpb. plantarum* LA15 was intermediately sensitive and the other five strains were resistant. All the strains were found to be sensitive to Tetracycline and Gentamicin, which are antibiotics with a wide spectrum of action. Similarly, almost all strains were found susceptible to Novobiosin (NV) and broad‐spectrum Chloromphenicol (C) ( *B. simplex* BA5 intermediate to C; *Lpb. plantarum* LA15 intermediate to NV). Three of the strains tested in this study (*B. simlex, B. mojevensis, B. pumilus*) were sensitive to Ciprofloxacin, which is known to be one of the antibiotics that have an antibacterial effect by disrupting the functions of DNA gyrase or topoisomerase enzymes. In contrast, the other strains ( *B. clausii* BA8, *B. subtilis* BA11, *B. cereus* BA13) were moderately sensitive. *Lpb. plantarum* LA15 was found resistant only to Rifampicin and Ciprofloxacin. In addition to this result, it is noteworthy that *B.pumilus* BA10 is particularly sensitive to the antibiotics used in the present study, except Oleandomycin. In this scope of study, some probiotic *Bacillus* strains exhibited varying degrees of resistance to various antibiotics. Similarly, it was stated that different *Bacillus* strains have different antibiotic susceptibility (Kharwar, Bazaz, and Dandekar 2022). The antibiotic susceptibility and GI tract conditions results for *B. clausii* BA8 and *B. subtilis* BA11 were found to be similar to those of *Lpb. plantarum* LA15.

**TABLE 3:** Susceptibility of all‐tested strains against antibiotics.

Name of the Antibiotics/Microorganisms	P (10 U)	AM (10 μg)	NV (30 μg)	C (30 μg)	OL (15 μg)	E (15 μg)	TE (30 μg)	CN (10 μg)	T (30 μg)	RA (5 μg)	F (300 μg)	CIP (5 μg)
*B. simlex* BA5	R	R	S	I	S	S	S	S	S	S	I	S
*B. clausii* BA8	R	R	S	S	R	R	S	S	S	S	S	I
*B. mojevensis* BA3	S	R	S	S	I	S	S	S	R	R	S	S
*B. pumilus* BA10	S	S	S	S	I	S	S	S	S	S	S	S
*B. subtilis* BA11	R	R	S	S	R	I	S	S	R	R	I	I
*B. cereus* BA13	R	R	S	S	R	S	S	S	R	R	S	I
*Lpb. plantarum* LA15	S	I	I	S	I	S	S	S	I	R	I	R

*Note:* Antibiotics including; Penicillin G (P), Ampicillin (AM), Novobiocin (NV), Erythromycin (E), Tetracycline (TE), Gentamicin (CN), Oxytetracycline (T), Rifampicin (RA), Nitrofurantoine (F), Ciprofloxacin (CIP), Chloramphenicol (C), Oleandomycin (OL).

### 3.5 Safety and Probiotic Properties With WG

The diagnosis of these two strains through WG analysis and summary data of WG sequencing are shown in Table 4. Genome‐based identification according to average nucleotide identity (ANI) calculation, two strains were assigned to *B. clausii* BA8 and *B. subtilis* BA11 with values of 98.1% and 97.8%, respectively, compared with the reference *B. clausii* (now *Shouchella clausii*) ATCC 700160 and *B. subtilis* 168 strain. Genome annotation of the strains was predicted using Prokka. *B.clausii* BA8 genome consisted of 110 contigs with a total length of 4.498.248 bp, GC content of 44%, 4477 genes, including 4399 protein‐coding sequences (CDS), 75 tRNAs, 12 rRNAs, and 1 tmRNA. *B. subtilis* BA11 genome consisted of 26 contigs totaling 4.215.606 bp, GC content of 43%, 4124 genes, including 4029 protein‐coding sequences (CDS), 83 tRNAs, 11 rRNAs, and 1 tmRNA. No plasmid sequences were found in either genome. In the current study, we screened the genomes of *B. clausii* BA8 and *B. subtilis* BA11 for various probiotic trait‐related genes and ARG to determine their probiotic functions at the genomic level.

**TABLE 4:** General genome features of *Bacillus clausii* BA8 and *Bacillus subtilis* BA11.

	*B.clausii* BA8	*B.subtilis* BA11
Contigs	110	26
GC Content (%)	44	43
Contig L50	12	2
Plasmids	0	0
Genome size (bp)	4,498,248	4,215,606
Contig N50	110,191	1,043,746
CDS	4399	4029
rRNA	12	11
tRNA	75	83
tmRNA	1	1
Genbank Accession	NZ_CP140150.1	NC000964.3

In this study, 16 genes were identified in *B. clausii* BA8, and 21 genes were identified in *B. subtilis* BA11. Probiotic gene screening is listed in Tables 5 and 6. In addition to genes encoding adhesion‐related proteins such as lipoprotein signal peptidase (*lspA*), elongation factor Tu (*tuf*), putative glycosyltransferase (*epsE*), sortase A (*srtA*), maltose phosphorylase (*mapA*), two genes essential for biofilm formation (*slpA, slpB*), lactate utilization proteins (*lutA, lutB, lutC*, and *lutP*) were also identified in *B. subtilis* BA11. *B. clausii* BA8 and *B. subtilis* BA11 were found to encode nine genes related to heat shock regulators (*hrcA, ctsR, dnaK, htpG, htpX, grpE, clpE, clpX*, and *clpP*), which are essential in intracellular protein aggregation and membrane stabilization, thus enabling them to withstand high temperatures (Lopez and Makhatadze 2000). In addition, *B. clausii BA8* contains two genes (*cspA, cspC*), while *B. subtilis* BA11 contains three genes (*cspB, cspC, and cspD*) that encode cold shock proteins, which are crucial for survival at low temperatures. CSP family genes are activated in several Bacillus strains to mitigate the harmful effects of cold stress (Su et al. 2020). Therefore, *B. clausii* BA8 and *B. subtilis* BA11 likely function similarly in cold stress adaptation.

**TABLE 5:** List of probiotic marker genes identified in the *Bacillus clausii* BA8 genomes.

Gene	Function	Gene Nos.
Adhesion		
*epsE*	Putative glycosyltransferase	1
*epsL*	Putative sugar transferase	1
*LDL*	L‐lactate dehydrogenase	1
*lldR*	Putative L‐lactate dehydrogenase operon regulatory protein	1
*lutP*	L‐lactate permease	1
*lacF*	Lactose transport system permease protein	1
*lacG*	Lactose transport system permease protein	1
*lspA*	Lipoprotein signal peptidase	1
*gpr*	L‐glyceraldehyde 3‐phosphate reductase—	1
*tpiA*	Triosephosphate isomerase (TIM)	1
*gapA*	Glyceraldehyde‐3‐phosphate dehydrogenase	2
*bgaP*	Beta‐galactosidase	1
*srtA*	Sortase A	1
*eno*	Enolase	1
*pgi*	Glucose‐6‐phosphate isomerase	1
*tuf*	Elongation factor Tu	1
Temperature		
Cold stress		
*cspA*	Cold shock protein	1
*cspC*	Cold shock protein	1
Heat stress		
*hrcA*	Heat‐inducible transcription repressor	1
*htpX*	Heat shock protein	3
*htpG*	Chaperone protein	1
*dnaK*	Chaperone protein	1
*grpE*	Chaperone protein GrpE	1
*clpE*	ATP‐dependent Clp protease ATP‐binding subunit	1
*clpX*	ATP‐dependent Clp protease ATP‐binding subunit	1
*clpP*	ATP‐dependent Clp protease proteolytic subunit	1
*ctsR*	Transcriptional regulator	1
Acid stress		
*nhaC*	Na(+)/H(+) antiporter NhaC	2
Bile tolerance		
*ppaC*	Manganese‐dependent inorganic pyrophosphatase	1
Antioxidant		
*katE*	Catalase	1
*fnr*	Ferredoxin‐NADP reductase	3
*bsaA*	Glutathione peroxidase	1
*ndh*	NADH dehydrogenase‐like protein	1
*npr*	NADH peroxidase	1
*nox*	NADH oxidase	2
*tpx*	Thiol peroxidase	1
*msrA*	Peptide methionine sulfoxide reductase	2
*msrB*	Peptide methionine sulfoxide reductase	1
*mntA*	Manganese‐binding lipoprotein	1
*mntB*	Manganese transport system ATP‐binding protein	1
*mntC*	Manganese transport system membrane protein	1
*mntD*	Manganese transport system membrane protein	1
*mntH*	Divalent metal cation transporter	1
*mntR*	HTH‐type transcriptional regulator	1
*sodA*	Superoxide dismutase [Mn]	2
*sodC*	Superoxide dismutase [Cu‐Zn]	1
Antimicrobial peptide		
*nisB*	Nisin biosynthesis protein	1
*nisC*	Nisin biosynthesis protein	1

**TABLE 6:** List of probiotic marker genes identified in the *Bacillus subtilis* BA11 genomes.

Gene	Function	Gene Nos.
Adhesion		
*slpA*	Biofilm‐surface layer protein A	1
*slpB*	Putative biofilm‐surface layer protein B	1
*lspA*	Lipoprotein signal peptidase	1
*mapA*	Maltose phosphorylase	1
*tuf*	Elongation factor Tu	1
*tpiA*	Triosephosphate isomerase (TIM)	1
*gapA*	Glyceraldehyde‐3‐phosphate dehydrogenase	2
*eno*	Enolase	1
*pgi*	Glucose‐6‐phosphate isomerase	1
*epsD*	Putative glycosyltransferase	1
*epsE*	Putative glycosyltransferase	1
*epsF*	Putative glycosyltransferase	1
*epsG*	Transmembrane protein EpsG	1
*epsH*	Putative glycosyltransferase	1
*epsI*	Putative pyruvyl transferase	1
*epsJ*	Putative glycosyltransferase	1
*epsK*	Putative membrane protein	1
*epsL*	Putative sugar transferase	1
*epsM*	Putative acetyltransferase	1
*epsN*	Putative pyridoxal phosphate‐dependent aminotransferase	1
*epsO*	Putative pyruvyl transferase	1
*lutA*	Lactate utilization protein A	1
*lutB*	Lactate utilization protein B	1
*lutC*	Lactate utilization protein C	1
*lutP*	L‐lactate permease	1
Temperature		
Cold stress		
*cspB*	Cold shock protein	1
*cspC*	Cold shock protein	1
*cspD*	Cold shock protein	1
Heat stress		
*hrcA*	Heat‐inducible transcription repressor	1
*htpX*	Heat shock protein	3
*dnaK*	Chaperone protein	1
*dnaJ*	Chaperone protein	1
*grpE*	Chaperone protein	1
*clpE*	ATP‐dependent Clp protease ATP‐binding subunit	1
*clpX*	ATP‐dependent Clp protease ATP‐binding subunit	1
*clpP*	ATP‐dependent Clp protease proteolytic subunit	1
*ctsR*	Transcriptional regulator	1
Acid stress		
*nhaC*	Na(+)/H(+) antiporter *NhaC*	1
*nhaX*	Stress response protein	1
Bile tolerance		
*ppaC*	Manganese‐dependent inorganic pyrophosphatase	1
*yocM*	Salt stress‐responsive protein	1
Antioxidant		
*katE*	Catalase	1
*fnr*	Ferredoxin–NADP reductase	3
*tpx*	Thiol peroxidase	1
*trxA*	Thioredoxin	1
*trxB*	Thioredoxin reductase	1
*ndh*	NADH dehydrogenase‐like protein	1
*msrA*	Peptide methionine sulfoxide reductase	1
*msrB*	Peptide methionine sulfoxide reductase	1
*mntA*	Manganese‐binding lipoprotein	1
*mntB*	Manganese transport system ATP‐binding protein	1
*mntC*	Manganese transport system membrane protein	1
*mntD*	Manganese transport system membrane protein	1
*mntH*	Divalent metal cation transporter	2
*mntR*	HTH‐type transcriptional regulator	1
*sodA*	Superoxide dismutase [Mn]	1

The *nhac* gene was identified from the *B. clausii* BA8 and *B. subtilis* BA11 genomes. This gene encodes sodium‐proton antiporters and is associated with the ability to adapt to an acidic environment. (Wai Liew et al. 2007). The *ppaC* encoding inorganic pyrophosphatase gene was identified in both strains for bile salt resistance. The *yocM* gene was also identified in *B. subtilis* BA11. This gene protects *Bacillus* strains against heat and salt stress (Hantke et al. 2019). Oxidative stress is a condition caused by the action of oxidizing compounds or reactive oxygen species (ROS), and this stress is associated with environmental factors (Duport et al. 2021; Kandasamy et al. 2022). For organisms such as *Bacillus* species, adaptation to an aerobic lifestyle is based on coping with oxidative stress effectively (Wu et al. 2019). *B. subtilis* BA11, which has a complete thioredoxin system (tpx, trxA, trxB), can eliminate ROS and reactive nitrogen species at a higher reaction rate by donating electrons to thiol‐dependent peroxidases. The *tpx* was detected in *B. clausii* BA8. In addition, the glutathione peroxidase (*gpx*) gene in *B. clausii* BA8 can detoxify hydrogen peroxide and lipid peroxyl radicals by regulating the protein dithiol/disulfide balance (Kandasamy et al. 2022).

Methionine sulfoxide reductase genes (*msrA, msrB*) were identified in both strains. It has been reported that these genes reduce oxidized methionine in the protein structure. (Duport et al. 2021). NADH oxidase/peroxidase and catalase may play a direct role in the degradation of hydrogen peroxide and ROS (Kandasamy et al. 2022). Genes encoding antioxidant systems were detected in *B. subtilis* BA11 *ndh*, *B. clausii* BA8 *ndh, npr*, and *nox*. Superoxide dismutase (*sodA, sodC*), manganese transport systems (*mnt*A‐D), and catalase (*katE*), which are an essential group of enzymes that reduce oxidative stress in the cell and combat free radicals, were detected in both strains (Sachla et al. 2024).

The ARGs identified in *B. clausii* BA8 and *B. subtilis* BA11, are presented in Table 7. The most prevalent genes were detected as *bla*_BCL‐1_, *erm, ant*(*4*')*‐lb* on *B. clausii* BA8. Among these *bla*_BCL‐1_, which is effective against the β‐Lactam group, *mphK*, which is effective against the macrolide group, and the *erm* gene, which is effective against erythromycin. Macrolide resistance and the *erm* gene constitute an advantage when co‐administered with oral macrolides, enabling the probiotic to be maintained in the intestine thanks to this resistance (Bozdogan, Galopin, and Leclercq 2004). Furthermore, studies have indicated that the *B. clausii* strain may possess the *erm* (Bozdogan, Galopin, and Leclercq 2004) and *aadD2* (Bozdogan et al. 2003) genes that could not be transferred to resistance genes through conjugation.

**TABLE 7:** Antibiotic resistance genes with NCBI, Card, Argannot and Megares in *B. subtilis* BA11 and *B. clausii* BA8.

Closest species (ANI^a^%)	Resistance gene	Identity%	Coverage %	Database	Accession	Affecting antibiotic class	References
*B. clausii* BA8 (98.1%)	*bla*_BCL‐1_	96.91	100	NCBI	NG_051318.1	Beta‐lactam	(Girlich et al. 2007)
	erm	97.87	100	NCBI	NG_047777.1	Macrolide	(Tartık 2023)
	*ant*(*4*')*‐lb*	94.16	100	NCBI	NG_047392.1	Aminoglycoside	(Sabtcheva et al. 2003)
*B. subtilis* BA11 (97.8%)	*mphK*	99.24	100	NCBI	NG_065846.1	Macrolide	(Wash et al. 2022)
	*vmlR*	98.48	100	NCBI	NG_063831.1	Lincosamide	(Takada et al. 2022)
	*rphC*	82.52	99.39	NCBI	NG_063825.1	Rifampin	(Liu et al. 2021)
	*aadK*	98.95	100	NCBI	NG_047379.1	Aminoglycoside	(Sulthana, Lakshmi, and Madempudi 2019)
	*satA*	100	100	NCBI	NG_064662.1	Streptothricin F	(Burckhardt and Escalante‐Semerena 2017)
	*tet*(L)	100	100	NCBI	NG_048204.1	Tetracycline	(Wei and Bechhofer 2002)
	*lmrB*	98.61	100	Argannot	NG_047912101–1540:1440	Multidrug Efflux Pump	(Yoshida et al. 2004)
	*tmrB*	98.48	100	Megares	MEG_7262	Tunicamycin	(Noda et al. 1992)
	Blt	99.16	100	Megares	MEG_1464	Multidrug Efflux Pump	(Woolridge et al. 1997)
	*ykkC*	97.05	100	Card	AL009126:1376516–1,376,855	Multidrug Efflux Pump	(Tshipamba, Lubanza, and Mwanza 2020)
	*ykkD*	98.43	100	Card	AL009126:1376854–1,377,172	Multidrug Efflux Pump	(Jack et al. 2000)
	*rphB*	80.41	97.78	Card	KX531052.1:0–2655	Rifamycin	(Liu et al. 2021)

^a^ Species identification based on whole genome average nucleotide identity (ANI).

In the present study, the following genes were identified in *B. subtilis* BA11: *mphK, vmlR, rphC, rphB, aadK, satA, tet*(*L*), *lmrB, tmrB, Blt, ykkC*, and *ykkD*. Among these genes, the *tet*(L) gene was the most notable because this strain was sensitive to tetracycline in the disk diffusion method. These genes on *B. subtilis* are related to various cellular processes, including sporulation, biofilm formation, and stress responses of bacterial physiology (Milton and Cavanagh 2023). Probiotic *Bacillus* strains usually harbor numerous genes that regulate their adaptation to the GI tract in stress responses (temperature, oxidative stress, pH, Ox‐Bile, etc.) (Khatri, Sharma, and Subramanian 2019; Ghelardi et al. 2022). These findings reveal that *B. clausii* BA8 and *B. subtilis* BA11 may be suitable for the adaptation abilities of the gastrointestinal system with their resistance to multiple stress conditions.

## 4 Conclusion

This study aimed to determine a more comprehensive probiotic profile for potential new probiotic strains of *Bacillus* spp. For this purpose, a safety assessment of potential probiotic *Bacillus* strains was conducted using accurate and reliable identification with MALDI‐TOF MS, whole genome analysis, and evaluation of phenotypic characteristics like biogenic amine formation. Specifically, the potential probiotic strains *B. clausii* BA8 and *B. subtilis* BA11 exhibited satisfactory results comparable to *Lactiplantibacillus*, known as reference strains. They demonstrated resistance to GI tract conditions and essential characteristics like auto‐aggregation and antibiotic resistance abilities. Within the scope of the study utilized, phenotypic analysis combined with WG analysis to determine antibiotic resistance characteristics, classify *Bacillus* species, and predict genes for safety purposes. These analyses provided valuable insights into the probiotic potential of these strains. The findings suggest that *B. clausii* BA8 may be a promising probiotic alternative to LABs, commonly used as probiotics due to their high commercial and biotechnological potential. However, further investigations are necessary to assess its potential health benefits and safety profile in vivo, especially in animal models.

## Author Contributions

**Nursel Söylemez‐Milli:** conceptualization (equal), data curation (equal), formal analysis (equal). **Pelin Ertürkmen:** conceptualization (equal), data curation (equal), formal analysis (equal). **Duygu Alp Baltakesmez:** conceptualization (equal), data curation (equal), formal analysis (equal).

## Ethics Statement

This research does not require ethical approval.

## Conflicts of Interest

The authors declare no conflicts of interest.

## Data Availability

The data used to support the conclusions of this research are accessible from the corresponding author upon reasonable request.
